# Grounding evidence in experience to support people-centered health services

**DOI:** 10.1007/s00038-018-1180-9

**Published:** 2018-12-12

**Authors:** Anna Dion, Lawrence Joseph, Vania Jimenez, Alessandro Carini Gutierrez, Amal Ben Ameur, Emilie Robert, Neil Andersson

**Affiliations:** 1 Department of Family Medicine, McGill University, 5858 chemin de la Côte des Neiges, Montreal, QC H3S 1Z1 Canada; 2 Department of Epidemiology, Biostatistics and Occupational Health, McGill University, Montreal, QC Canada; 3 CARE Canada, Ottawa, ON Canada; 4 SHERPA (Recherche – Immigration – Société) - Centre de recherche du CSSS de la Montagne, Montreal, QC Canada; 5 ICARES, Montreal, QC Canada; 6 Centro de Investigación de Enfermedades Tropicales (CIET), Universidad Autónoma de Guerrero, Acapulco, Mexico

“*Making health care truly universal requires a shift from health systems designed around diseases and health institutions towards health systems designed around and for people.*” (Zsuzsanna Jakab, WHO Regional Director for Europe) (James et al. 2018)

## Introduction

Evidence-informed and equity-oriented public health policy and practice require that people’s voices, especially those less heard, be central to decision-making in public health (Serrant-Green 2011). Stakeholder engagement is particularly urgent in the context of health inequities, where perspectives of those who carry the greatest burden of inequities are often poorly reflected in published literature (Serrant-Green 2011). Decision-makers in public health need robust and locally relevant tools that take account of both biomedical and cultural understandings of health and that support people’s participation in planning, implementation and evaluation (Napier et al. 2014).

Leveraging several well-established tools from participatory research, systems science and Bayesian analysis, under a critical realist philosophy, we present a novel approach to knowledge synthesis, called the *Weight of Evidence*. This approach pushes conventional boundaries of who (or what) constitutes health service expertise through the formal inclusion of experiential knowledge from patients and/or communities, care providers and resource decision-makers, together on even footing with epidemiological studies (Borda 1996; Midgley 2000). This method unfolds in five steps:

1. A conventional mixed methods synthesis of the research literature summarizes what is known about an outcome of interest, representing this knowledge as a map;
2. Independently, stakeholders generate cognitive maps that identify and weight factors they believe influence the outcome;
3. Update the literature-based map with stakeholder knowledge using Bayesian analysis;
4. Suggest explanations of *how* social, economic and organizational contexts contribute to outcomes prioritized in cognitive maps; stakeholders adjust these explanations according to their experience; and
5. Stakeholders develop recommendations accordingly.

In this publication, we outline the *Weight of* Evidence process, highlighting some of the key insights from our pilot work addressing inequities in perinatal health in Canada, while a full description of our methodological development results is forthcoming. *Weight of Evidence* proved an excellent way to engage meaningfully with divergent perspectives, creating space for multiple and complex ways of understanding health and health services.

### Mapping evidence

**Fig. 1:**
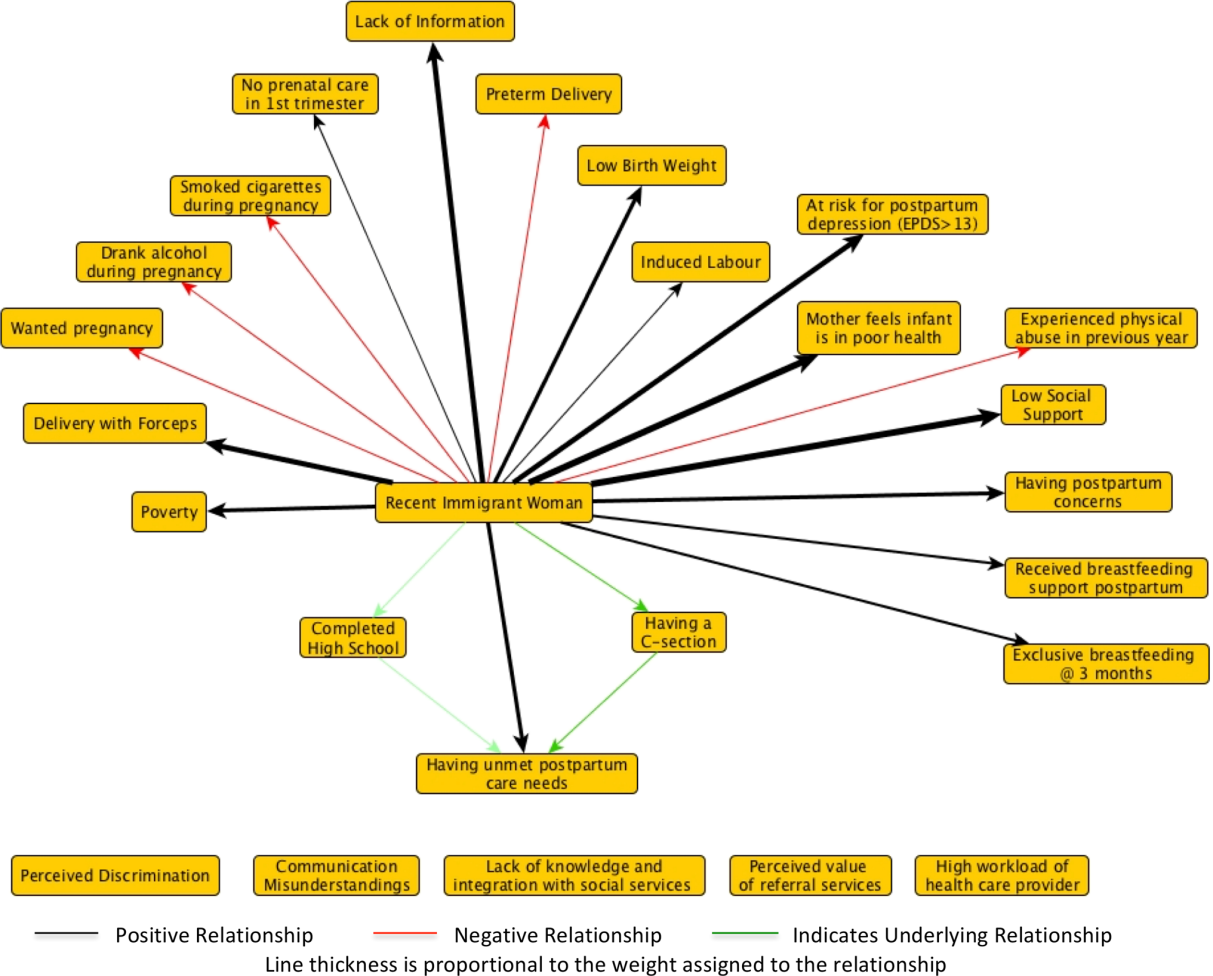
Fuzzy cognitive map of available literature on unmet postpartum care needs among recent immigrant women in Canada. EPDS is the Edinburgh Postnatal Depression Scale. A score greater than 13 on the EPDS is interpreted as probable depression (Cox et al. 1987) (Canada, 2016)

Step 1 follows existing guidelines to support comprehensive mixed methods evidence syntheses, pooling effect estimates when appropriate using standard meta-analyses techniques (Pluye and Hong 2014). We converted all effect estimates to odds ratios and transformed them into a common scale (− 1 to + 1) (Andersson et al. 2017). We then summarized findings in a concept map where nodes in the map represent themes from qualitative studies or independent variables from quantitative studies, and the strength of the arcs connecting nodes describe the effect estimates (Özesmi and Özesmi 2004; Giles et al. 2008). In our demonstration case, we focused on unmet postpartum care needs among recent immigrant women as an important health inequity in Canada (Gagnon et al. 2013). Our concept map also included evidence from the broader literature on perinatal health outcomes and experiences of recent immigrant women in Canada, as shown in Fig. 1.

### Co-producing evidence

For Step 2, determining who needs to be at the table is often driven by what expertise is considered relevant (Midgley 2000). This is of particular importance in matters of health inequities, as those who live with the everyday effects of vulnerability bring relevant expertise on their access to care and their ability to maintain their health and well-being, yet are often excluded from decision-making processes (Borda 1996). Thoughtful and extensive consideration of who to engage, and how, has important implications for how the process unfolds. In our demonstration case, we recruited stakeholders for accessibility and their ability to contribute to the understanding of the issue as either a healthcare provider or social support to recent immigrant women in a large Canadian city. Informed by published evidence, stakeholders are guided through the development of their own cognitive maps, describing factors they believe influence the outcome (Özesmi and Özesmi 2004; Giles et al. 2008). Stakeholders then assign a weight or perceived importance, on a scale of 1 through 5 and direction of effect (+ ve or − ve), to each relationship in their updated map.

In our demonstration case, stakeholder-identified factors were notably more actionable than those identified in the literature. Service providers and patient representatives focused less on conventional individual “risk factors” (e.g., education or specific health behaviors) and more on the support systems around women throughout the perinatal period. This illustrated how including stakeholder knowledge as a complement to published literature can broaden both the problem definition and the menu of interventions.

Cognitive maps that account for interdependence between factors can act as a decision aid for complex processes like clinical care, where artificially isolating associations within a de facto network or results chain can diminish the contextual understanding and relevance of decisions (Napier et al. 2014). Step 3 accounts for this interdependence first by normalizing stakeholder-assigned weights to the same − 1 to + 1 scale used for the literature-based maps, creating a comparable relative measure of the importance of each factor to our outcome of interest: 0 indicating no importance and + 1 (or − 1) indicating great importance in determining the outcome. A transitive closure algorithm (ProbTC), allows weights between factors (scale of 0–1) to be analyzed using probability theory, (Niesink et al. 2013) as has been done in other areas of medicine and public health (Giles et al. 2008; Andersson et al. 2017). This algorithm adjusts each weight to account for all other factors in the map, and highlights walks, or underlying relationships between factors, identifying possible priorities in addressing the outcome (Niesink et al. 2013).

**Fig. 2:**
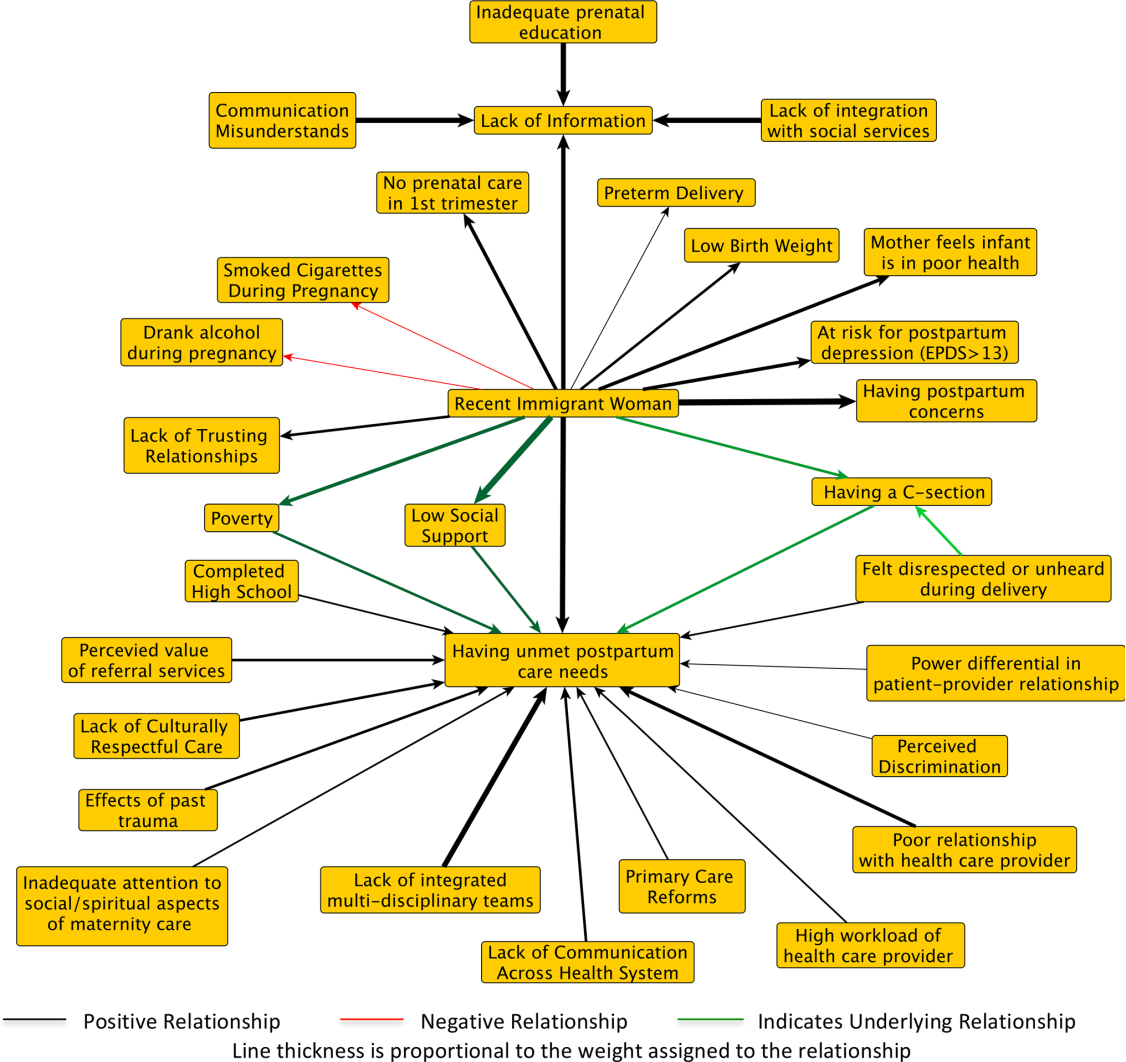
Fuzzy cognitive maps of the literature updated by family physicians. EPDS is the Edinburgh Postnatal Depression Scale. A score greater than 13 on the EPDS is interpreted as probable depression (Cox et al. 1987) (Canada, 2016)

**Fig. 3:**
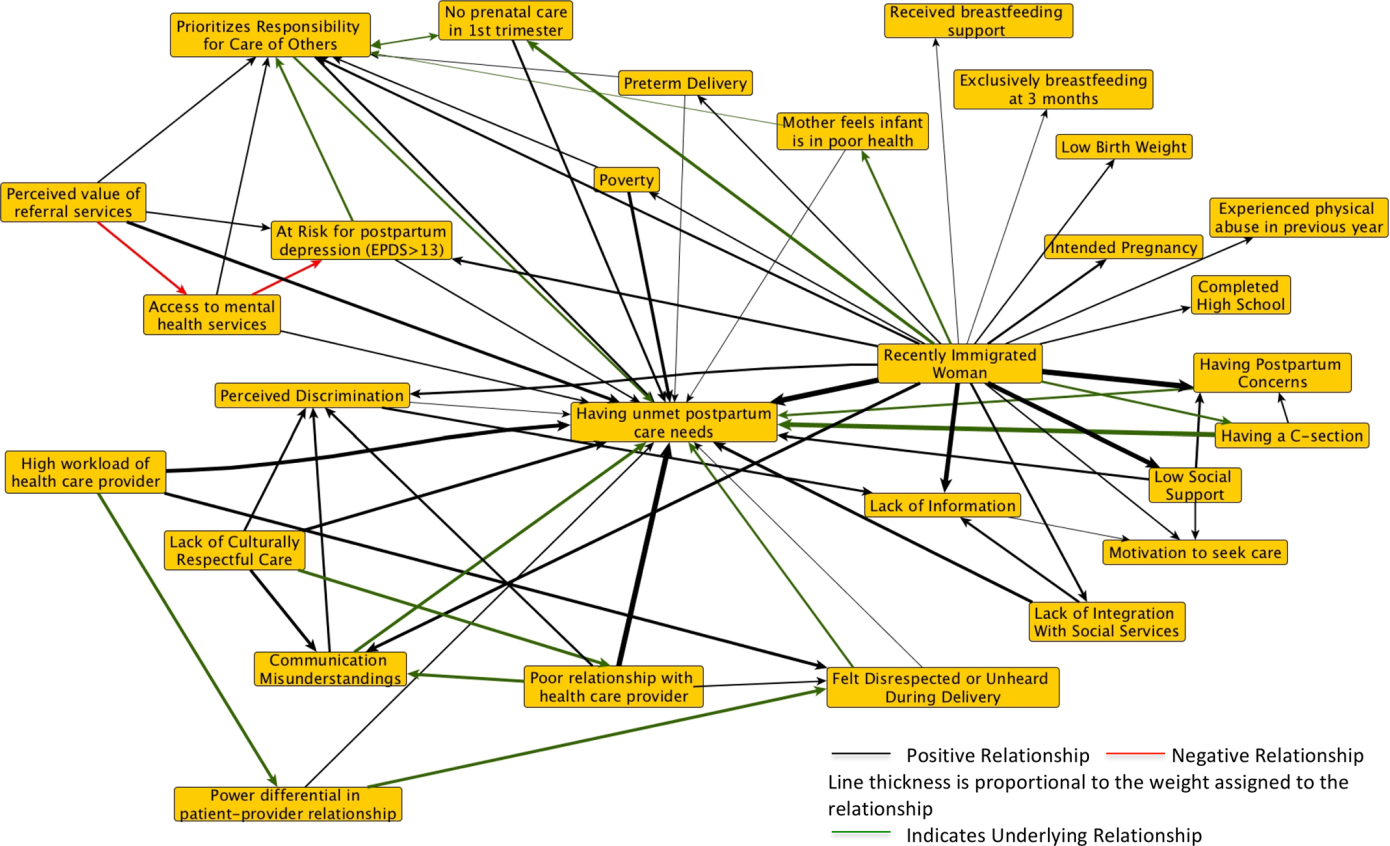
Fuzzy cognitive maps of the literature updated by patient representatives. EPDS is the Edinburgh Postnatal Depression Scale. A score greater than 13 on the EPDS is interpreted as probable depression (Cox et al. 1987) (Canada, 2016)

To bring these different perspectives in conversation with one another, we drew on Bayesian analysis as a formal method to integrate stakeholder perspectives with published literature. Conventional Bayesian analysis elicits prior weights from experts by asking how likely they consider the occurrence of an event to be (Gelman et al. 2013). Our approach instead asks patients and other stakeholders how important they consider each factor to be to the outcome, what (relative) weight would they place on this factor. Describing both stakeholder views and published evidence using weights normalized to the same (− 1, + 1) scale, Bayesian analysis combines what is *known* about a relationship with observed *data* about that same relationship, by calculating a posterior distribution using Bayes’ theorem (Goldstein 2006; Gelman et al. 2013). This also allows for a formal accounting of the uncertainty around both epidemiological data and stakeholder perspectives, highlighting differences in perspectives both within and between knowledge sources. Each updating of published evidence with stakeholder knowledge produces a new architecture, as weights are reinforced where there are areas of agreement between stakeholders and published literature and diminished where there are areas of disagreement (Goldstein 2006; Kruschke 2015). Figures 2 and 3 show the published evidence on unmet postpartum care needs updated by family physician perspectives and patient representatives, respectively.

### Patient-centered improvement strategies

Step 4 requires that we understand cognitive maps as conceptual, not probabilistic models (Mingers 2005). Along with the narratives that accompany their construction, they show how stakeholders make sense of their experience in the context of evidence from the literature. Here, explanatory power draws on critical realist philosophy, where explanatory accounts point to *how* social, economic and organizational contexts contribute to outcomes prioritized in the literature or in stakeholder maps (Pawson 2000; Bhaskar 2008). Stakeholders are then asked to adjust these possible explanations to coincide with their experience. This is especially important when working with marginalized communities, a setting where theories and explanations generated outside the community may reinforce erroneous stereotypes (Tuck 2008). Bringing diverse perspectives together can balance often implicit assumptions within clinical practice, health services and policies with patient experience and understanding (Harris et al. 2016). Our demonstration case showed how the lack of supportive relationships for marginalized women influenced perinatal health and highlighted how specific policy or organizational structures can contribute to unresponsive care.

Step 5 focuses on the identification of care recommendations. Engaging stakeholders in the explanatory analysis in the previous steps creates space not only for different forms of knowledge about how a particular system works but also shifts the realm of possible improvement strategies (Midgley 2000).

### Methods to support more responsive health services

Moving toward more people-centered health services requires that we take better account of how people’s understandings of determinants of poor health intersect with conventional biomedical evidence (Napier et al. 2014). Yet few methods within primary healthcare research preserve divergent perspectives, ending up instead homogenizing and losing the richness within difference (Keller 1992). *Weight of Evidence* presents a rigorous and transparent approach to unpack differences, to identify how and when these differences arise and with what consequences.

We share this work as an invitation to include methodological innovations as part of our collective response to calls for more people-centered health systems (James et al. 2018). Citizens, particularly those carrying the greatest burden of health inequities, need to have a stronger voice in the planning and implementation of their health care and the systems meant to support it. Participatory methods that are both robust and transparent are key to getting us there.
